# Resin-based adhesives, composites, and luting agents: Investigation of article citations and altmetrics

**DOI:** 10.1590/1807-3107bor-2025.vol39.041

**Published:** 2025-04-04

**Authors:** Fernanda LAUER, Rodrigo Rohenkohl SILVA, Leticia Regina SARTORI, Kauê COLLARES, Rafael SARKIS-ONOFRE, Clóvis FAGGION JUNIOR, Rafael Ratto de MORAES

**Affiliations:** (a) Universidade Federal de Pelotas – UFPel, Graduate Program in Dentistry, Pelotas, RS, Brazil.; (b) Atitus Educação, Graduate Program in Dentistry, Passo Fundo, RS, Brazil.; (c) University Hospital Münster, Department of Periodontology and Operative Dentistry, Münster, Germany.

**Keywords:** Dental Materials, Bibliometrics, Altmetrics, Journal Article

## Abstract

In this study, citations, altmetric scores, and field-normalized impact of articles investigating resin-based adhesives, luting agents, or restorative composites were investigated. Articles published in 2019 on resin-based dental materials indexed in Scopus were searched and assessed by independent investigators. Data collected in 2023 included several article variables and, as outcomes, citation in Scopus and Google Scholar, Altmetric Attention Scores (AAS), and Field-Weighted Citation Impact (FWCI). Data were analyzed using stepwise backward quasi-Poisson regression models (p < 0.05). A total of 707 articles were included, which evaluated restorative composites (58.3%), adhesives (27.2%), and luting agents (19.5%). The majority of corresponding authors were from Asia/Oceania (42.2%), with publications mainly subscription-based (54%) and lacking international collaboration (68.5%). Only 1.4% reported conflicts of interest, and 47.7% did not disclose sponsorship. Median citations were 7 in Scopus and 13 in Google Scholar, whereas the median FWCI was 1. The majority of articles had an AAS of zero. Multivariate analysis showed study sponsorship type and journal CiteScore influenced citations, while COI and the author’s continent impacted AAS and FWCI, respectively. Articles on luting agents were less likely to receive citations. The report of conflict of interest was associated with approximately 18 times higher AAS values. This study emphasizes the significance of the type of resin-based material, journal CiteScore, authors’ continent, and type of sponsorship in affecting citations, visibility, and impact of scientific articles. Research on luting agents may need better dissemination strategies for increased visibility. The substantial effect of COI presence underscores the importance of transparency.

## Introduction

Scientific knowledge dissemination relies on peer-reviewed articles, the recognition of which is often measured by the number of citations they receive. New research builds on previous studies, expanding subjects and furthering knowledge. Factors influencing literature choice are complex and not always tied to their relevance or quality.^1^ Social networks enable rapid content exchange, with around 5 billion active users globally.^2^ Few academic scientists use these tools, although promotion strategies can increase article citations and visibility.^3^ This shift created a need for metrics such as altmetrics, which measure social web impact, tracking interactions to gauge social impact. The Altmetric platform calculates Altmetric Attention Scores (AAS).^4^

Numerous bibliometric studies have examined fields such as the top 100 cited articles on dental sealants,^5^ endodontic therapy in primary teeth,^6^ and periodontology.^7^ In dental materials, studies have assessed the most cited articles on dentin adhesives,^8^ dental ceramics,^9^ and those in dental materials journals.^10^ While these studies identify characteristics of highly cited articles, they do not determine factors contributing to high citation rates. For example, Yu et al.^11^ found that open-access articles had significantly higher citation counts and AAS over seven years compared with subscription articles. This raises the question of which additional variables lead to a scientific article receiving more citations or online attention.

Identifying factors that influence citation counts is fundamental to improving research visibility, impact, and mapping trends in research areas.^12^ Resin-based materials are among the materials most researched and used in dentistry and have continuously evolved since the advent of adhesion technology. However, it remains unclear how articles on resin-based materials perform in terms of bibliometric and altmetric data and whether the type of resin-based material influences citation frequency. Factors such as the specificity of the material, authors’ country, or international collaboration may influence citations or online attention. Understanding these factors could provide a more precise view of the literature on these dental materials, how they are perceived by the community, and offer a comprehensive perspective of the publishing landscape. Additionally, there is potential for exploring more recent article-level, field-normalized impact metrics in dental research, such as the Field-Weighted Citation Impact (FWCI) and its interplay with other metrics.

The aim of this scientometric cross-sectional study was to investigate the number of citations, AAS, and FWCI of articles investigating resin-based adhesives, luting agents, and restorative composites, published in dental journals. Factors associated with citations, online attention, and field-normalized impact were analyzed. The null hypothesis was that no differences would be observed among articles on adhesives, luting agents, or restorative composites.

## Methods

### Study design

The study protocol was registered (DOI 10.17605/OSF.IO/GZTJU) and this report follows the STROBE guidelines for cross-sectional studies,^13^ with adaptations explained. All papers in the field of dentistry evaluating resin-based materials and published in journals indexed in Scopus during 2019 were included. Table 1 presents all the variables of interest that were collected from each publication and their sources of information. Dependent variables included citation-based measures as outcomes, whereas independent variables encompassed aspects related to the publications, journals, and authors.

**Table 1:** Variables of interests and their sources of information.

Variables	Type	Source
Independent variables		
Resin-based material (adhesive/composite/luting agent)	Categorical	Article
Number of authors	Numerical	Article
Continent of corresponding author	Categorical	Article
Publication access type (open access/subscription)	Categorical	Scopus
Presence of international collaboration (yes/no)	Categorical	Article
Conflict of interest (COI) (none reported/unclear/reported)	Categorical	Article
Sponsorship (none/non-profit/for-profit/mixed/unclear)	Categorical	Article
Journal	Categorical	Scopus
CiteScore 2023	Numerical	Scopus
Journal Impact Factor (JIF) 2023	Numerical	Journal Citation Reports
Dependent variables		
Article citations	Numerical	Scopus / Google Scholar
Field-Weighted Citation Impact (FWCI)	Numerical	Scopus
Altmetric Attention Score (AAS)	Numerical	Altmetric.com

### Search strategy and eligibility criteria

A structured search was performed in Scopus to identify studies reporting original research on resin-based dental materials, as shown in the protocol. This database was selected for its comprehensive coverage of dental journals and the quantity of information it provides on citations and metrics. The search was refined considering year (2019), area (dentistry), document type (article), publication stage (final), source (journal), and language (English). The year 2019 was selected because it was the last year before the influence of the COVID-19 pandemic, in order to avoid an over-publication of scientometric studies and dental topics related to the disease, in addition to allowing a period for citations to accumulate.^14^ A single year was chosen to avoid differences in citation timeframes from different years.

For this study, resin-based materials were either adhesives, which promote bonding to different surfaces; restorative composites, materials with a polymer matrix highly filled with inorganic particles; or luting agents, which are composites with lower filler loading, and are used for fixing structures. Articles were included if they focused on the specific material as the main study outcome. For example, studies in the adhesives category needed to evaluate the effect, influence, impact, behavior, or performance of one or more dental bonding agents, even if the adhesive was associated with other materials. If a study aim was to evaluate a light-curing unit, it was included provided that the main outcome was related to one of the eligible materials. Studies that could not be designated as research on adhesives, composites, or luting agents were excluded: i.e., glass ionomer cement, varnish, silane, acrylic resin, and polymer-containing ceramic. All types of studies were included except opinions, letters, and editorials.

### Screening

The search results were exported to a web app (Rayyan, Cambridge, MA, USA), where two investigators (FL and RRS) assessed the articles independently to reduce bias. The investigators were previously trained using a random sample of 50 articles from the search (randomizer.org). The investigators applied the eligibility criteria and a more experienced researcher (RRM) checked the results. In all cases, a consensus was reached. After training, the researchers assessed the titles and abstracts, consulting full texts in case of doubt. In cases of disagreement, consensus was reached after discussion, and if necessary, the experienced investigator’s opinion was decisive.

### Data collection

Data were collected, recorded, and entered into an Excel spreadsheet by one evaluator (FL), and all the data were checked by a second evaluator (RRS). Categorical variables were categorized by one evaluator (RRS), and checked by the other evaluator (FL). To avoid updates on metrics, the quantitative variables were collected in a single week in July 2023 by a group of trained researchers. For each article, citations were collected in Scopus and Google Scholar, in addition to FWCI (Scopus) and AAS (Altmetric bookmarklet installed in a web browser).

Scopus and Google Scholar citations, and AAS, were considered primary outcomes because they provide measures of the attention gained by the articles in different scenarios, thus offering a broader view of impact.^15^ The FWCI was treated as a secondary outcome. The FWCI is a field-weighted metric that is calculated by using the ratio of the total citations received by the article to the total citations expected based on the average of the subject field.^16^ An FWCI of 1 indicates that the article performed at the global average, while values above or below 1 indicate more or fewer citations than the global average.

The resin-based materials analyzed in this study are frequently used in combination, such as pairing an adhesive with a resin composite. In many instances, there may be material overlap, with each occurrence of different materials being counted. The variable was treated as dichotomous, for instance, whether an adhesive was present (yes/no). International collaboration was noted whenever two or more countries were represented in the authors’ affiliations. If an author had more than one affiliation, only the first was recorded. If the corresponding author information was unavailable, the last author was designated as the corresponding author. The author’s continents were categorized into Africa, the Americas, Asia/Oceania, and Europe. The COI and sponsorship could be noted as none, reported, or unclear, i.e., when it was not explicitly stated whether it was present or the information was missing. Sponsorship was classified as follows: non-profit sponsors, which included government, university, hospital, research institute, and charitable foundations; for-profit sponsors encompassed companies; mixed sponsorship involved both non-profit and for-profit entities; and unclear sponsorship pertained to cases where it was not evident whether the sponsor was a non-profit or for-profit organization.^17^ Donation of materials was considered a type of sponsorship and was classified according to the type of sponsor.

### Data analysis

Data were analyzed using descriptive statistics to estimate the absolute and relative frequencies of the variables of interest, along with their respective 95% confidence intervals. Analyses were conducted for each of the four dependent variables. Stepwise backward quasi-Poisson regression models, accounting for overdispersion, were used to assess associations between the independent variables and citations in Scopus, citations in Google Scholar, AAS, and FWCI. To be included in the final model, p < 0.2 was considered; in the final model, statistical significance was considered if p < 0.05. No sensitivity analysis was conducted. Prevalence Ratios were obtained to quantify the effect of the independent variables. Jamovi software 2.4.8.0 (Sydney, Australia) was used.

## Results

Initially, 4,109 records were identified, from which 785 full-text articles were assessed for eligibility, and 707 articles were ultimately included for data extraction (Figure).

**Figure:**
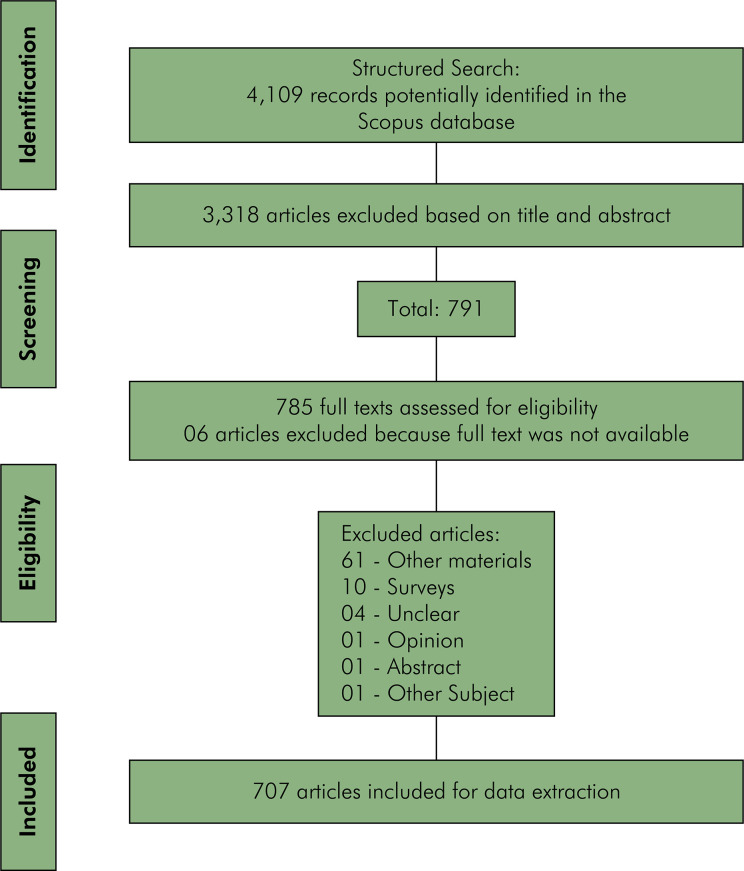
Flowchart of the search and eligibility process.

### Article characteristics and citations

From the sample of 707 papers, 58.3% evaluated restorative composites, 27.2% evaluated adhesives, and 19.5% evaluated luting agents (Table 2). These percentages account for the overlap between the resin-based materials. The most prevalent continent for corresponding authors was Asia/Oceania (42.2%). The most common type of publication access was subscription-based (54%), and the majority of articles did not involve international collaboration (68.5%). A very small fraction of the articles reported the presence of COI (1.4%), and 47.7% of the articles did not disclose any form of sponsorship. The most frequent journals in this sample were Dental Materials, Operative Dentistry, and Clinical Oral Investigations. The median number of authors per article was 5, with the highest number being 13. Not all journals had CiteScore and JIF metrics available; however, among the articles from journals with CiteScore, the median was 4.2, with the highest being 13.4. Among the articles from journals with JIF, the median was 3.0, with the highest being 7.6. The median number of citations per article in the Scopus database was 7, with the highest being 134. In the Google Scholar database, the median number of citations was nearly double that of Scopus, at 13, with the highest being 232. The median FWCI was 1, with the highest being 15. The majority of articles had an AAS of zero, with the highest AAS found being 44.

**Table 2:** Characteristics of dependent and independent variables collected from the articles (n = 707).

Variables	n (%)
Resin-based dental material	
Restorative composite	412 (58.3)
Adhesive	192 (27.2)
Luting agent	138 (19.5)
Continent of the corresponding author	
Asia/Oceania	298 (42.2)
Americas	256 (36.2)
Europe	143 (20.2)
Africa	10 (1.4)
Publication access type	
Subscription-based	382 (54)
Open access	325 (46)
International collaboration	
Yes	223 (31.5)
No	472 (68.5)
COI	
None reported	415 (58.7)
Unclear	282 (39.9)
Reported	10 (1.4)
Sponsorship	
None/Unclear	337 (47.7)
Non-profit	249 (35.2)
For-profit	64 (9.1)
Mixed	57 (8.1)
Journal*	
Dental Materials	55 (7.8)
Operative Dentistry	46 (6.5)
Clinical Oral Investigations	38 (5.4)
The Journal of Adhesive Dentistry	37 (5.2)
Dental Materials Journal	34 (4.8)
Journal of Prosthodontics	30 (4.2)
Journal of Dentistry	28 (4.0)
The Journal of Prosthetic Dentistry	23 (3.3)
Journal of Contemporary Dental Practice	20 (2.8)
Others	396 (56)
Numerical variables	Median (25^th^–75^th^)
Number of authors	5.0 (4.0–6.0)
CiteScore 2023 (n = 696)	4.2 (2.6–6.3)
JIF 2023 (n = 518)	3.0 (2.2–4.0)
Article citations in Scopus	7.0 (3.0–15.0)
Article citations in Google Scholar (n = 702)	13.0 (6.0–26.0)
FWCI	1.0 (0.0–2.0)
AAS (n = 669)	0.0 (0.0–0.0)

*Journals with at least 20 articles in the sample. COI: conflict of interest; FWCI: Field-Weighted Citation Impact; JIF: Journal Impact Factor.

### Multivariate analysis of citations in Scopus and Google Scholar

Table 3 presents the results of the regression analysis for citations in Scopus. In the adjusted model, associations were found for the following variables: CiteScore, sponsorship, and luting agent. For CiteScore, each one-unit increase was associated with a 14% higher prevalence of citations. Articles with for-profit sponsorship or mixed sponsorship had 43% and 50% higher prevalence of citations, respectively, compared with articles with none or unclear sponsorship. In contrast, articles investigating resin-based luting agents were 26% less likely to receive citations in Scopus compared with articles that did not investigate this type of resin-based material.

**Table 3:** Results of the regression analysis among independent variables and article citations in Scopus (n = 707).

Variable	Crude		Adjusted	
	PR (95%CI)	p-value	PR (95%CI)	p-value
Number of authors	1.10 (1.06–1.15)	<.001	1.01 (0.97–1.05)	0.621
CiteScore 2023 (n = 696)	1.19 (1.16–1.22)	<.001	1.14 (1.02–1.27)	0.021*
JIF 2023 (n = 518)	1.28 (1.20–1.35)	<.001	0.95 (0.77–1.18)	0.694
Adhesive (Ref. No)		0.457		
Yes	1.07 (0.89–1.29)	0.455		
Resin composite (Ref. No)		0.245		
Yes	1.11 (0.93–1.32)	0.246		
Luting agent (Ref. No)		0.040		0.005
Yes	0.79 (0.63–0.99)	0.045	0.74 (0.60–0.91)	0.006*
Continent of corresponding author (Ref. Asia/Oceania)		<.001		0.351
Americas	1.41 (1.16–1.71)	<.001	1.14 (0.94–1.39)	0.160
Europe	1.71 (1.37–2.12)	<.001	1.20 (0.96–1.50)	0.099
Africa	0.92 (0.40–2.12)	0.849	1.27 (0.41–2.94)	0.621
International collaboration (Ref. No)		<.001		0.089
Yes	1.66 (1.41–1.96)	<.001	1.15 (0.97–1.36)	0.089
Publication access type (Ref. Subscription)		<.001		0.163
Open access	0.60 (0.50–0.72)	<.001	0.88 (0.73–1.05)	0.167
Sponsorship (Ref. None/Unclear)		<.001		0.005
Non-profit	1.58 (1.31–1.91)	<.001	1.16 (0.95–1.42)	0.127
For-profit	1.79 (1.35–2.33)	<.001	1.43 (1.08–1.88)	0.01*
Mixed	2.36 (1.82–3.04)	<.001	1.50 (1.16–1.93)	0.002*
COI (Ref. Not reported)		0.068		0.835
Reported	0.87 (0.39–1.94)	0.742	1.02 (0.45–2.01)	0.957
Unclear	1.21 (1.02–1.44)	0.024	1.05 (0.88–1.25)	0.548

*Statistical significance. PR: Prevalence ratio; CI: Confidence interval; JIF: Journal Impact Factor; COI: Conflict of interest. The R-squared value in this analysis was 0.224, indicating that the variables in the regression model explain approximately 22.4% of the variability in Scopus citation counts.

Table 4 shows the results of the regression analysis for citations in Google Scholar. In the adjusted model, statistical significance was observed for two of the same three variables in Scopus, namely sponsorship and luting agent, except for CiteScore which was borderline (p = 0.056). Articles with for-profit and mixed sponsorship were 36% and 52% more likely to receive citations, respectively. In addition, articles investigating luting agents had a 24% lower prevalence of citations in Google Scholar, compared with studies that did not include this type of resin-based material.

**Table 4:** Results of the regression analysis among independent variables and article citations in Google Scholar (n = 702).

Variable	Crude		Adjusted	
	PR (95% CI)	p-value	PR (95% CI)	p-value
Number of authors	1.08 (1.04–1.12)	< 0.001	0.99 (0.95–1.04)	0.932
CiteScore 2023 (n = 696)	1.16 (1.13–1.19)	< 0.001	1.11 (0.99–1.12)	0.056
JIF 2023 (n = 518)	1.20 (1.13–1.28)	< 0.001	0.95 (0.77–1.17)	0.659
Adhesive (Ref. No)		0.551		
Yes	0.94 (0.78–1.14)	0.553		
Resin composite (Ref. No)		0.023		0.462
Yes	1.22 (1.03–1.44)	0.024	1.07 (0.63–0.93)	0.464
Luting agent (Ref. No)		0.028		0.040
Yes	0.78 (0.62–0.98)	0.033	0.76 (0.59–0.98)	0.043*
Continent of corresponding author (Ref. Asia/Oceania)		< 0.001		0.283
Americas	1.42 (1.17–1.72)	< 0.001	1.18 (0.96–1.44)	0.100
Europe	1.62 (1.31–2.00)	< 0.001	1.17 (0.93–1.47)	0.169
Africa	1.33 (0.67–2.62)	0.406	1.57 (0.61–3.32)	0.284
International collaboration (Ref. No)		< 0.001		0.086
Yes	1.55 (1.32–1.83)	< 0.001	1.16 (0.97–1.38)	0.086
Publication access type (Ref. Subscription)		< 0.001		0.599
Open access	0.70 (0.59–0.83)	< 0.001	0.95 (0.79 - 1.14)	0.6
Sponsorship (Ref. None/Unclear)		< 0.001		0.008
Non-profit	1.44 (1.20–1.72)	< 0.001	1.11 (0.90–1.36)	0.317
For-profit	1.71 (1.29–2.22)	< 0.001	1.36 (1.02–1.78)	0.029*
Mixed	2.25 (1.75–2.87)	< 0.001	1.52 (1.17–1.96)	0.001*
COI (Ref. Not reported)		0.509		
Reported	1.35 (0.64 – 2.82)	0.427		
Unclear	1.08 (0.91 – 1.28)	0.344		

*Statistical significance. PR: Prevalence ratio; CI: Confidence interval; JIF: Journal Impact Factor; COI: Conflict of interest. The R-squared value in this analysis was 0.165, indicating that the variables in the regression model explain approximately 16.5% of the variability in Google Scholar citation counts.

### Multivariate analysis of AAS and FWCI

Table 5 presents the results of the regression analysis for AAS. In the adjusted model, associations were observed for the continent of the corresponding author and COI. Articles from corresponding authors from the Americas were 2.25 times more likely to have a higher AAS score than corresponding authors affiliated with Asia/Oceania. The report of COI was associated with approximately 18 times higher expected AAS than articles that did not report COI. Table 6 presents the results of the regression analysis for FWCI. In the adjusted model, associations were again found for the continent of the corresponding author and COI. Articles from corresponding authors from the Americas had 25% more expected FWCI counting (Europe was borderline, p = 0.058). For-profit and mixed sponsorship was associated with a 39% and 53% increase in the prevalence of FWCI counting, respectively.

**Table 5:** Results of the regression analysis among independent variables and Altmetric Attention Scores – AAS (n = 669).

Variable	Crude		Adjusted	
	PR (95% CI)	p-value	PR (95% CI)	p-value
Number of authors	1.08 (0.92–1.26)	0.338		
CiteScore 2023 (n=696)	1.21 (1.10–1.34)	< 0.001	1.25 (0.82–1.92)	0.294
JIF 2023 (n=518)	1.27 (1.01–1.61)	0.041	0.77 (035–1.71)	0.528
Adhesive (Ref. No)		0.431		
Yes	1.32 (0.64–2.60)	0.424		
Resin composite (Ref. No)		0.973		
Yes	1.01 (0.50–2.07)	0.973		
Luting agent (Ref. No)		0.265		
Yes	0.58 (0.18–1.44)	0.296		
Continent of corresponding author (Ref. Asia/Oceania)		0.003		0.116
Americas	3.54 (1.64–7.67)	0.001	2.25 (1.09–4.65)	0.028*
Europe	2.61 (1.07–6.39)	0.034	2.03 (0.85–4.84)	0.108
Africa	1.07e-6 (0.00–∞)	0.990	1.83e-6 (0.00–∞)	0.992
International collaboration (Ref. No)		0.367		
Yes	1.40 (0.66–2.86)	0.360		
Publication access type (Ref. Subscription)		0.050		0.560
Open access	0.51 (0.24–1.00)	0.058	0.82 (0.43–1.57)	0.564
Sponsorship (Ref. None/Unclear)		0.101		0.201
Non-profit	2.23 (1.09–4.79)	0.031	1.95 (0.95–3.98)	0.066
For-profit	2.72 (0.91–7.21)	0.053	0.82 (0.22–2.99)	0.766
Mixed	1.60 (0.38–4.99)	0.456	1.11 (0.37–3.29)	0.847
COI (Ref. Not reported)		< 0.001		<0.001
Reported	15.8 (4.94–56.1)	< 0.001	17.7 (4.03–77.6)	< 0.001*
Unclear	0.92 (0.43–1.96)	0.840	0.87 (0.45–1.64)	0.662

*Statistical significance. PR: Prevalence ratio; CI: Confidence interval; JIF: Journal Impact Factor; COI: Conflict of interest. The R-squared value in this analysis was 0.167, indicating that the variables in the regression model explain approximately 16.7% of the variability in AAS.

**Table 6:** Results of the regression analysis among independent variables and Field-Weighted Citation Impact – FWCI (n = 707).

Variable	Crude		Adjusted	
	PR (95% CI)	p-value	PR (95% CI)	p-value
Number of authors	1.10 (1.06–1.15)	<.001	1.01 (0.96–1.06)	0.637
CiteScore 2023 (n=696)	1.18 (1.14–1.22)	< 0.001	1.05 (0.93–1.19)	0.374
JIF 2023 (n=518)	1.24 (1.16–1.32)	<0.001	1.07 (0.86–1.34)	0.527
Adhesive (Ref. No)		0.585		
Yes	1.06 (0.86–1.30)	0.584		
Resin composite (Ref. No)		0.604		
Yes	1.05 (0.87–1.27)	0.605		
Luting agent (Ref. No)		0.244		
Yes	0.86 (0.68–1.11)	0.251		
Continent of corresponding author (Ref. Asia/Oceania)		< 0.001		0.147
Americas	1.50 (1.21–1.86)	< 0.001	1.25 (1.00–1.56)	0.042*
Europe	1.71 (1.35–2.17)	< 0.001	1.27 (0.99–1.64)	0.058
Africa	1.01 (0.42–2.44)	0.979	1.38 (0.50–3.81)	0.528
International collaboration (Ref. No)		< 0.001		
Yes	1.66 (1.39–2.00)	< 0.001	1.17 (0.97–1.42)	0.100
Publication access type (Ref. Subscription)		< 0.001		
Open access	0.63 (0.52–0.76)	< 0.001	0.93 (0.76–1.14)	0.493
Sponsorship (Ref. None/Unclear)		< 0.001		0.015
Non-profit	1.61 (1.31–1.97)	< 0.001	1.19 (0.95–1.50)	0.116
For-profit	1.80 (1.34 - 2.43)	< 0.001	1.39 (103–1.89)	0.028*
Mixed	2.47 (1.87–3.25)	< 0.001	1.53 (1.15–2.04)	0.003*
COI (Ref. Not reported)		0.818		
Reported	0.75 (0.31–1.85)	0.545		
Unclear	0.99 (0.82–1.20)	0.967		

*Statistical significance. PR: Prevalence ratio; CI: Confidence interval; JIF: Journal Impact Factor; COI: Conflict of interest. The R-squared value in this analysis was 0.133, indicating that the variables in the regression model explain approximately 13.3% of the variability in FWCI.

## Discussion

This study is the first to explore the factors affecting the citations and online visibility of international dental research articles on resin-based materials and the interplay among different article variables and research metrics. Our findings indicated that a higher CiteScore, the corresponding author’s continent, sponsorship type, and the reporting of conflicts of interest significantly influenced citation rates and related metrics. Overall, the citation patterns of articles in the Scopus and Google Scholar databases were similar and appear to be equally usable, although the number of citations were higher in Google Scholar. The analysis also revealed that among the resin-based dental materials, research on luting agents tended to receive fewer citations. Consequently, the null hypothesis was rejected.

The finding that articles focusing on luting agents tended to have fewer citations was unique and unprecedented in the literature, making it difficult to compare with previous studies. A recent study analyzing the composition of dental products reported that among all commercially available resin-based materials, only 13.5% were intended for luting procedures.^18^ An older study, conducted before the widespread use of electronic databases, found that luting agents accounted for less than 10% of total citations in that sample of articles.^19^ A possible explanation for our findings could be that luting agents do not have the same visibility or marketing approaches as those applied to adhesives and restorative materials, which are seen as being more central to the durability of dental treatments. Luting agents might be viewed as an auxiliary or accessory to other materials such as indirect restorations or glass-fiber posts. The reasons behind the lower citation rates of articles on luting agents need to be further investigated. Furthermore, despite the trend of fewer citations for articles investigating luting agents, the FWCI metric did not show a significant difference in impact for this group of articles compared with adhesives and restorative composites.

Relative to the journal-based metrics, an increase in CiteScore was generally associated with a higher expected number of citations for articles. Thus, CiteScore appears to better able to predict citation potential for resin-based dental materials research compared with JIF. This occurred despite the 2023 CiteScore being used for articles published in 2019, which may be explained by the fact that CiteScore covers a longer citation window (4 years) than JIF (2 years). A case study with journals from six different subject fields suggested that CiteScore may effectively measure relative citation impact within the same field.^20^ However, an analysis indicated that the updated CiteScore formula, which includes early citations, biased results in favor of journals with high early citation rates.^21^ Another study found that CiteScore was less affected by the amount of editorial material in a journal compared with JIF.^22^ However, these metrics should not be seen as competitors; rather, a combined use of metrics provides a broader view of article citation impact. It is also important to reinforce that metrics are not indicators of the quality of studies or journals.

Another notable finding was that articles with for-profit or mixed sponsorship had higher expected citations and FWCI. While it is inconceivable to suggest that authors should seek higher impact through mixed or for-profit sponsorship due to the risk of diverting research from public health priorities, this result prompts reflection on the underlying reasons. Studies sponsored by for-profit organizations may introduce more publication bias or research spin, which could favor positive results and lead to more citations. Approximately one-third of randomized trials in high-impact dental journals with non-significant outcomes showed spin in their abstracts,^23^ although sponsorship type did not influence this. Another study reported that spin-containing trials had approximately 1.5 times more citations than those without spin.^24^ However, since publication bias and spin were not the focus in this study, our findings cannot be explained from that perspective. Similarly, articles reporting COI had 18 times higher AAS than those without COI. This aligns with previous findings that publications reporting COI had greater odds of receiving for-profit sponsorship.^17^ Multiple factors and their interplay may affect citation and online attention metrics, leaving room for future studies on this topic.

In our sample, 54% of the articles were published as subscription-based, indicating that less than half of the research on resin-based dental materials is freely available to the scientific community. This matches the percentage found in a study on the prevalence of open access in dentistry.^25^ The cited study found that self-archiving (green open access) was more common than publisher-provided access (gold open access) and that open-access articles did not receive more citations than subscription-based articles. However, a subsequent study by the same group reported that open-access articles tended to have greater scientific and social impact in the long term, specifically seven years after publication.^11^ Moreover, 30% of the articles in our sample involved international collaboration, aligning with the findings of a previous study^26^ and slightly exceeding the findings of another study.^27^ However, citations or impact were not affected by the presence of international collaboration in our sample, which disagrees with a previous study.^28^ This finding might be related to the specific nature of the dental research considered in the present investigation rather than an effect representative of the entire dental literature.

Whereas AAS is a metric increasingly used in dental research to extend the impact of research by tracking online attention from different sources such as social media, news outlets, and policy, FWCI is an article-level impact metric that has not been widely explored in the dental literature. Most articles in our sample had an AAS of zero, contrasting with the median citations of 7 to 13 in other articles, indicating that the literature on resin-based dental materials generally does not receive much online attention. In healthcare, a study^29^ reported the opposite, some published articles might not receive any citations but can still have a significant social media impact. Other studies in dentistry found no association between AAS and citation counts.^8,30^ The median FWCI was 1, with the highest being 15, indicating considerable variability in the sample. If we consider FWCI as a valid impact metric, complemented by AAS, our findings suggest that literature on resin-based dental materials from authors in the Americas has a greater impact than that from other continents. Nevertheless, Asia/Oceania contributed the most articles to this sample, demonstrating the global scope of scientific publication in resin-based dental materials and the many contributors to its development. Furthermore, political issues relative to social media use in different countries could affect AAS.^31^

This study had some limitations. We attempted to provide an overview of dental research on resin-based materials, but we only considered one specific publication year, and the citation period was approximately four years. A larger sample, considering a longer timeframe, could reveal a different scenario. Moreover, although more citations and mentions are generally interpreted as positive, this study did not evaluate individual citations, which might include critiques or disagreements. Future studies could use qualitative citation analysis to gain deeper insights into the nature of citations. Nevertheless, our sample of over 700 articles appears to be sufficiently robust to provide relevant evidence on research related to resin-based dental materials, a relatively unexplored area in scientometrics. These findings can help in developing strategies to increase the visibility of articles, such as those on luting agents that seem to attract less attention. Further studies evaluating different periods, temporal influences on citations and metrics, and mapping collaboration networks could provide a comprehensive understanding of how research on resin-based dental materials has evolved. Moreover, a more detailed analysis of citations and online mentions would offer a dynamic view of research impact over time.

## Conclusion

This study emphasizes the significance of variables such as CiteScore, corresponding authors’ continent, and sponsorship type in affecting the visibility and impact of research on resin-based dental materials. The substantial effect of the presence of conflicts of interest underscores the importance of transparency in scholarly communication. Articles investigating luting agents tended to receive fewer citations and less visibility, suggesting a need for increased focus or different dissemination strategies for this topic.
